# MdNAC4 Interacts With MdAPRR2 to Regulate Nitrogen Deficiency-Induced Leaf Senescence in Apple (*Malus domestica*)

**DOI:** 10.3389/fpls.2022.925035

**Published:** 2022-06-30

**Authors:** Binbin Wen, Xingyao Gong, Qiuping Tan, Wenzhe Zhao, Xiude Chen, Dongmei Li, Ling Li, Wei Xiao

**Affiliations:** State Key Laboratory of Crop Biology, College of Horticulture Science and Engineering, Shandong Agricultural University, Taian, China

**Keywords:** apple, N deficiency, MdNAC4, MdAPRR2, leaf senescence

## Abstract

Nitrogen (N) is one of the important macronutrients in plants, and N deficiency induces leaf senescence. However, the molecular mechanism underlying how N deficiency affects leaf senescence is unclear. Here, we report an apple NAC TF, MdNAC4, that participates in N deficiency-induced leaf senescence. The senescence phenotype of apple leaves overexpressing *MdNAC4* was enhanced after N deficiency. Consistently, the chlorophyll content of transgenic leaves was significantly lower than that in the WT control leaves, the expression of chlorophyll catabolism-related genes (*MdNYC1*, *MdPAO*, and *MdSGR1*) was significantly higher than that in the WT controls, and the expression of chlorophyll synthesis-related genes (*MdHEMA*, *MdCHLI*, and *MdCHLM*) was significantly lower than that in the WT control leaves. Furthermore, MdNAC4 was found to directly activate the transcription of the chlorophyll catabolism-related genes *MdNYC1* and *MdPAO*. Additionally, MdNAC4 was proven to interact with MdAPRR2 proteins both *in vitro* and *in vivo*, and overexpression of *MdAPRR2* seemed to delay N deficiency-induced leaf senescence. Correspondingly, the chlorophyll loss of MdAPRR2-overexpressing (MdAPRR2-OE) lines was significantly lower than in WT control plants. Although downregulated, the expression of the chlorophyll synthesis-related genes *MdHEMA*, *MdCHLI*, and *MdCHLM* in the transgenic plants was more than twice that in the WT control plants. Taken together, our results enrich the regulatory network of leaf senescence induced by N deficiency through the interaction between MdNAC4 and MdAPRR2.

## Introduction

Leaf senescence is the last stage of leaf development and is characterized by the breakdown of plant organs and overwhelming catabolism (Woo et al., 2019). Through the leaf senescence process, the first visible phenotypic change is the color change of the leaf due to the breakdown of chlorophyll (Hörtensteiner, 2006, 2009). Leaf senescence involves the process of programmed cell death, in which the expression of some senescence-related genes is altered. Previous studies have identified transcription factors (TFs) that play a crucial role in the regulation of leaf senescence (Kim et al., 2018). These TFs include WRKY TFs (Besseau et al., 2012; Guo et al., 2017; Chen et al., 2017b; Yu et al., 2021), bHLH TFs (An et al., 2019; Hu et al., 2020), MYB TFs (Huang et al., 2015; Qi et al., 2015), and AP2/ERF TFs (Feng et al., 2016; Tan et al., 2018; Riester et al., 2019).

In particular, some TFs not only regulate leaf age-dependent senescence but also integrate developmental and environmental signals to regulate leaf senescence. The poplar NAC TF RD26 occurs as an alternative splicing variant (intron retention [IR]) and produces the truncated protein PtRD26^IR^. PtRD26IR inhibits leaf senescence, while normal PtRD26 promotes leaf senescence by regulating multiple senescence-related genes (Wang et al., 2021). RD26 is also involved in stress-induced leaf senescence and participates in chloroplast protein degradation by directly activating the chloroplast vesicle (CV) synthesis-related gene in the dark to accelerate leaf senescence (Kamranfar et al., 2018). The rice NAC TFs ORE1 and NAP regulate leaf senescence induced by plant hormones or stress by regulating the expression of senescence-related genes (Liang et al., 2014; Mao et al., 2017). NAC2 indirectly regulates leaf senescence by upregulating the expression of abscisic acid (ABA) synthesis-related genes (*NCED3* and *ZEP1*) and downregulating the expression of an ABA decomposition-related gene (*ABA8 ox1*; Singh et al., 2021). NAC33 can bind to the promoter of the chlorophyll degradation-related gene *SGR1* and regulate leaf senescence by promoting chlorophyll degradation (Kelly and Allan, 2021). These results suggest that NAC TFs play a key role in leaf senescence and directly or indirectly participate in leaf senescence through a variety of metabolic pathways.

N is an important macronutrient required for plant growth and development, and its deficiency can induce leaf senescence (Schulte auf'm Erley et al., 2007; Meng et al., 2016). NRT1.5, a xylem nitrate-loading transporter, is strongly induced by nitrate starvation. The current study shows that NRT1.5 plays an important role in perceiving nitrate deficiency signals and suppresses nitrate deficiency-induced leaf senescence by facilitating potassium accumulation in the leaves (Chen et al., 2012; Zhang et al., 2014; Meng et al., 2016). NRT1.7, a phloem nitrate transporter, is involved in nitrate remobilization from old leaves. During their vegetative growth, when plants encounter long-term N deficiency, NRT1.7 delays N deficiency-induced leaf senescence by transferring nitrate from old leaves to developing tissues (Fan et al., 2009). In addition, the *NITROGEN LIMITATION ADAPTATION* (*NLA*) gene plays an important role in regulating N deficiency-induced the leaf senescence (Kant et al., 2011; Park et al., 2018). NLA interacts with the NAC TF ORE1 in the nucleus and affects the process of leaf senescence by regulating the level of ORE1 proteins (Park et al., 2018). The results indicate that NAC TFs are involved in leaf senescence induced by N deficiency, but the potential role of *MdNAC4* in regulating leaf senescence induced by N deficiency has not been investigated in detail.

In recent years, research on leaf senescence has mostly focused on chlorophyll degradation. For example, *PHYTOCHROME-INTERACTING FACTOR (PIF)* family proteins PIF4/5 can directly or indirectly regulate the expression of chlorophyll catabolism-related genes (CCGs; including *NYC1*, *PAO*, and *NYE1*) and regulate darkness-induced leaf senescence (Sanchez et al., 2020). The maize NAC TF ZmNAC126 acts downstream of the ethylene metabolism pathway components and activates the expression of CCGs to accelerate leaf senescence (Yang et al., 2020). The MADS box family TF SOC1 directly inhibits the transcription of the CCGs *NYC1* and *PPH* to negatively regulate leaf senescence (Chen et al., 2017a). Other TFs, namely, ANAC016, ANAC019, ANAC046, ANAC055, ANAC072 and ANAC092, directly bind to the promoters of a set of major CCGs and positively regulate leaf senescence (Sakuraba et al., 2014, 2016; Qiu et al., 2015; Zhu et al., 2015; Oda-Yamamizo et al., 2016).

Chlorophyll degradation is often concomitant with chloroplast breakdown. In senescent leaves, the maintenance of chloroplasts plays an important role in counterbalancing leaf senescence (Rauf et al., 2013). Key elements in this process include Golden 2-like (GLK2) TFs that maintain chloroplast development (Waters et al., 2008). Studies have shown that the NAC TF ORE1 interacts with GLK TFs protein, and ORE1 antagonizes GLK transcriptional activity, resulting in chloroplast breakdown (Rauf et al., 2013). In addition, quantitative trait locus (QTL) studies have indicated that *APRR2* may be another candidate gene involved in the maintenance of chloroplast development (Brand et al., 2012). Expression analysis of *APRR2* showed that it was specifically expressed in immature fruit, and a nonsense mutation in *APRR2* caused the fruits to have a white color (Pan et al., 2013).

In the present study, it was found that the NAC TF MdNAC4 promoted leaf senescence induced by N deficiency by directly activating the transcription of the CCGs *MdNYC1* and *MdPAO*. We also identified a direct protein interaction between MdNAC4 and MdAPRR2. Overexpression of *MdAPRR2* seems to delay leaf senescence induced by N deficiency by increasing the expression of chlorophyll synthesis-related genes. In summary, we identified a novel NAC TF that participates in leaf senescence induced by N deficiency, which provided a new insight to study the molecular mechanisms of N deficiency-induced leaf senescence.

## Materials and Methods

### Plant Materials and Growth Conditions

The tissue-cultured plantlets of *Malus domestica* ‘GL-3’ were cultured under long-day conditions (14 h:10 h, light/darkness) at 24/22°C and the seedlings were subcultured once a month. The apple leaves used in the transient transformation experiments were taken from *M. domestica* ‘GL-3’ tissue culture seedlings. Detached leaves were placed on N-deficient plates under long-day conditions (14 h:10 h, light/darkness) at 22°C for 2 weeks. Sterilized tobacco (*Nicotiana benthamiana*) seeds were placed on Murashige and Skoog (MS) agar media at 4°C for 96 h, and then transferred to growth chamber. The tobacco plants were grown at 24/22°C under long-day conditions (14 h:10 h, light/darkness), and the leaves of tissue-cultured tobacco were used for genetic transformation, the leaves of substrate cultivated tobacco were used for dual-luciferase assays.

### RNA Extraction and Real-Time Quantitative PCR Analysis

Total RNA of the samples was extracted using an RNA prep Pure Plant Plus Kit (TIANGEN, Beijing, China). Afterward, single-stranded cDNA was synthesized using a cDNA Synthesis Kit (Vazyme, Nanjing, China). qRT-PCR analysis was performed using UltraSYBR Mixture (CW Biotech, Taizhou, China) in a CFX Connect™ Real-time System (Bio-Rad, CA, United States). Three technical replicates and three biological replicates were carried out for each sample. The primers for qRT-PCR were designed by NCBI Primer-BLAST and synthesized by Sangon Biotech Technology (Shanghai, China). The internal reference gene used is *MdActin* (*MD12G1140800*). The transcription-level analysis was performed using the comparative Ct (2^–ΔΔCt^) method. All the primer sequences used are listed in Supplementary Table S1.

### Generation of Transgenic Plant Materials

The specific sequence fragments of MdNAC4 were inserted into a tobacco rattle virus (TRV) vector to construct the antisense suppression expression plasmids. The full-length CDS of MdNAC4 were inserted into a pIR vector to construct the overexpression plasmids. The transient transformation of apple leaves was performed as described previously (An et al., 2019). Stable transgenic apple and tobacco seedlings were obtained by *Agrobacterium*-mediated genetic transformation. The coding DNA sequence (CDS) of MdAPRR2 was cloned into pBI121 vectors containing a GFP tag to construct overexpression plasmids. Transgenic plant materials were subsequently obtained as described previously (An et al., 2018; Zhao et al., 2020).

### Measurement of the Chlorophyll Content

The content of chlorophyll was determined as described above (Wen et al., 2019). In short, 0.2 g of senescent leaves were cut into small pieces and then immersed in 20 ml 96% ethanol for 24 h in the dark to extract the chlorophyll. The absorbance was measured at wavelengths of 470, 649 and 665 nm using a spectrophotometer (UV-2600 Shimadzu, Shanghai, China).

### Yeast One-Hybrid Assays

The full-length CDS of MdNAC4 were cloned into a pGADT7 vector. Similarly, the MdNYC1 and MdPAO promoter fragments were inserted into a pAbAi vector. Different combinations of recombinant plasmids were cotransformed into a yeast one-hybrid (Y1H) yeast strain. The yeast transformation product was then tested on media (SD/-Ura/-Leu) containing an optimal concentration of AbA.

### Yeast Two-Hybrid Assays

Fragments of MdNAC4^1–350 aa^, MdNAC4^1–146 aa^, MdNAC4^147–285 aa^, and MdNAC4^286–350 aa^ were inserted into pGBKT7 vector. The recombinant plasmid of different fragments was transformed into a Y2H yeast strain and cultured on SD/−Trp, SD-Trp/X-α-gal media at 30°C for 3–5 days. The full-length CDS of MdAPRR2 were cloned into pGADT7 vector. Different combinations of recombinant plasmids were subsequently cotransformed into a yeast two-hybrid (Y2H) yeast strain and cultured on SD/-Trp/-Leu media at 30°C for 3–5 days. Then, the yeast transformation product was transferred to SD/-Leu/-Trp/-His/-Ade and SD/-Leu/-Trp/-His/-Ade media containing X-α-gal.

### Dual Luciferase Assays

The promoter fragments of MdNYC1 and MdPAO were inserted into a pGreenII 0800-LUC vector to generate a reporter construct. The full-length CDS of MdNAC4 were then cloned into a pGreenII 62-SK vector to generate an effector construct. Transformation of *Agrobacterium tumefaciens* strain GV3101 with the recombinant plasmids. Tobacco leaves were injected with different *Agrobacterium* strain mixtures. The injected tobacco leaves were collected and sprayed with a D-luciferin sodium salt solution in the dark for 3–5 min. Using an *in vivo* imaging system to detect the fluorescence (Xenogen, Alameda, United States).

### Bimolecular Fluorescence Complementation Assays

The full-length CDS of MdNAC4 and MdAPRR2 were cloned into pSPYNE and pSPYCE vectors, respectively. Transformation of *A. tumefaciens* strain GV3101 with the recombinant plasmids. Onion epidermal cells were infected with the mixture of *A. tumefaciens* strains for 30 min. The infected onion epidermal cells were transferred to MS media as previously described (Chen et al., 2018). A laser scanning confocal microscope was used for fluorescence detection (Carl Zeiss, Oberkochen, Germany).

### Pull-Down Assays

The full-length CDS of MdNAC4 and MdAPRR2 were inserted into pET32a and pGEX-4 T-1 vectors, respectively. The MdNAC4-pET32a and MdAPRR2-pGEX-4 T-1 recombinant vectors were then transformed into *Escherichia coli* BL21 (TransGen, Beijing, China) and the recombinant proteins with HIS-tagged and glutathione S-transferase (GST)-tagged were induced by isopropyl-β-D-thiogalactopyranoside. The pull-down assays were performed using a HIS-tagged protein purification kit (CW Biotech, Taizhou, China) as previously described (Zhang et al., 2020). The sample proteins were collected after western blotting was performed and detected with anti-HIS and anti-GST antibodies (Abmart, Shanghai, China).

### Electromobility Shift Assays

The fusion proteins of MdNAC4-GST are induced by isopropyl-β-D-thiogalactopyranoside. Synthesis and labeling of the probes were performed by Sangon Biotech Co., Ltd. (Shanghai, China). The fusion protein and biotin-labeled probes were incubated at 24°C in binding buffer under dark conditions for 30 min. An unlabeled probe as a competition probe. After incubation, a protein loading buffer was added, and the polyacrylamide gel electrophoresis was performed (Thermo Scientific, San Jose, United States).

### Statistical Analyses

GraphPad Prism 6 software (GraphPad Software, La Jolla, CA, United States) was used for the drawing, and SPSS 19.0 software (SPSS, Chicago, IL, United States) was used for the statistical analyses. The significance of the differences was tested by *t*-tests or one-way (ANOVA; ^*^, *p* < 0.05, significant; ^**^, *p* < 0.01, extremely significant).

## Results

### Identification and Analysis of MdNAC4

**Figure 1:**
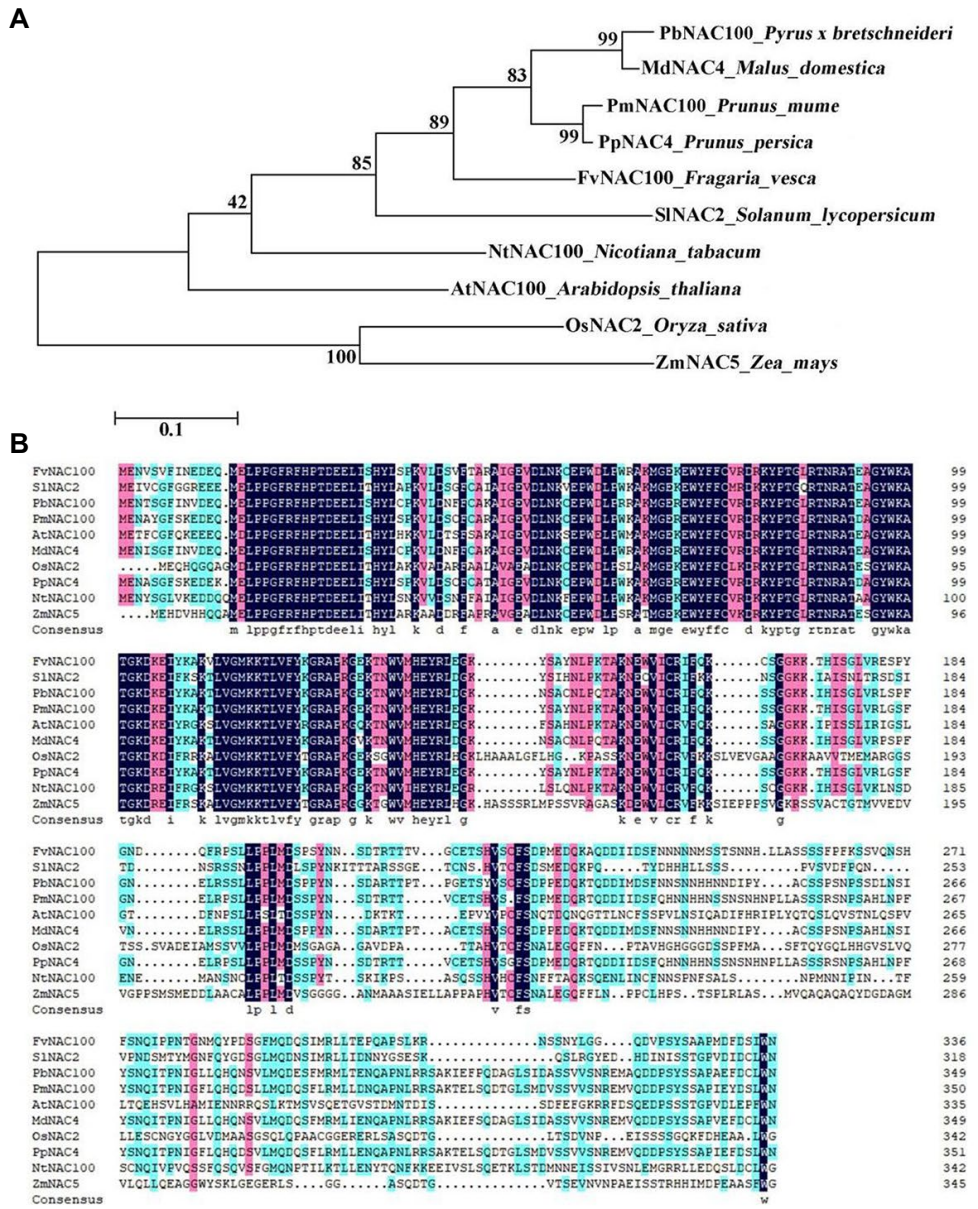
Phylogenetic tree analysis and amino acid sequence alignment of NAC4 proteins in different species. **(A)** Phylogenetic tree analysis of NAC4 proteins in different species; **(B)** Multiple alignments of NAC4 amino acid sequences in different species. (AtNAC100: NP_200951.1; NtNAC100: XP_016439814.1; OsNAC2: XP_015633922.1; ZmNAC5: PWZ29660.1; SlNAC2: NP_001316319.1; FvNAC100: XP_004304286.1; PpNAC100:ALK27823.1; PmNAC100: XP_008230794.1; PbNAC100: NP_001289216.1; MdNAC4: XP_008379376.2).

A phylogenetic tree was constructed from the NAC4 amino acid sequences of Arabidopsis_thaliana, Nicotiana_tabacum, Oryza_sativa, Zea_mays, Solanum_lycopersicum, Fragaria_vesca, Prunus_persica, Prunus_mume, Pyrus_bretschneideri, and Malus_domestica (Figure 1A). The results showed that MdNAC4 in the apple was the closest to PbNAC100 in white pear, followed by plum and peach. The amino acid sequence alignment analysis showed that the amino acid sequence of MdNAC4 was similar to PbNAC100, PmNAC100, and PpNAC4 (Figure 1B).

### Overexpression of *MdNAC4* Causes Early Senescence of Apple Leaves

**Figure 2:**
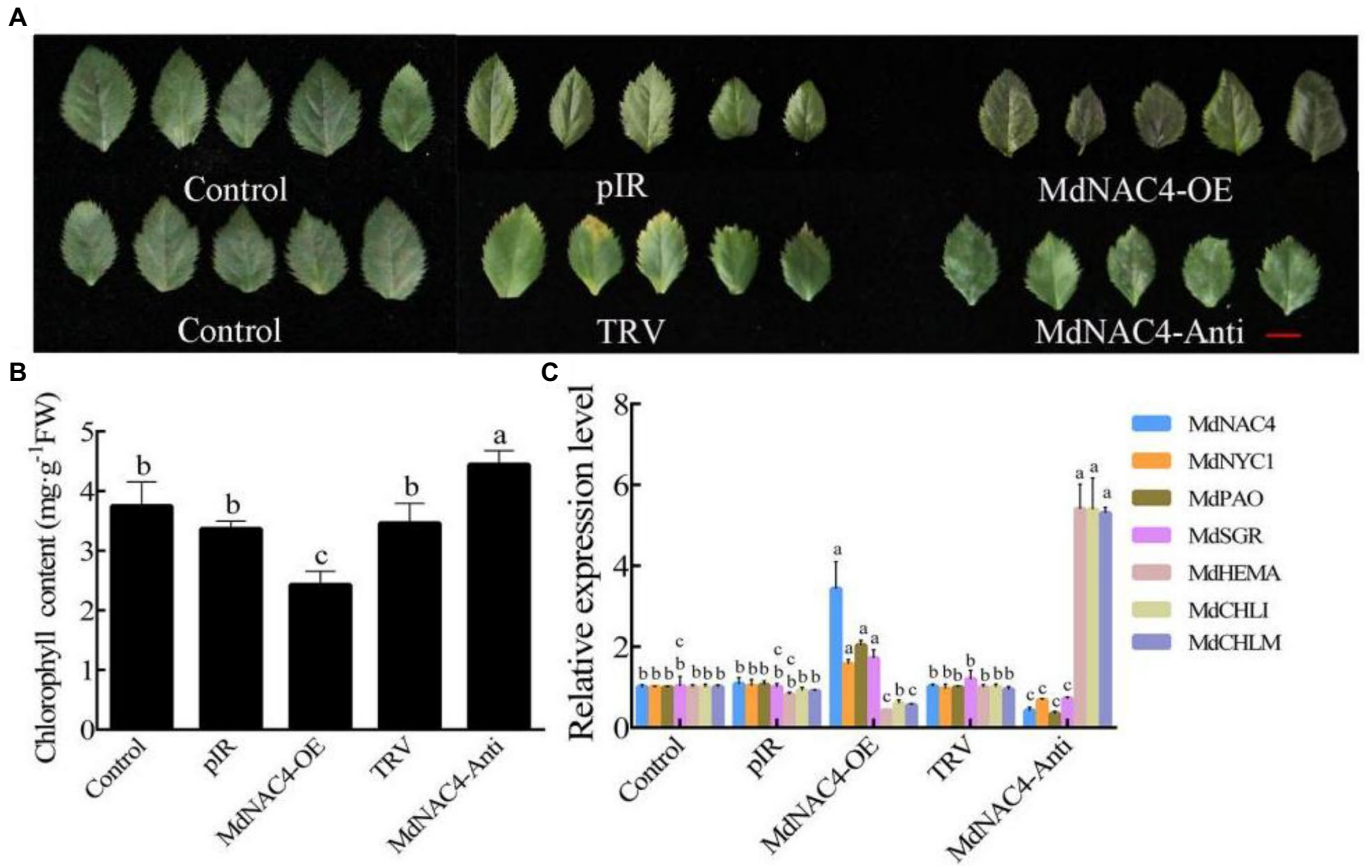
Overexpression of *MdNAC4* caused early senescence in apple leaves. **(A)** Phenotypes of apple leaves of WT (Control, pIR, TRV), MdNAC4 overexpression (MdNAC4-OE), and antisense MdNAC4 (MdNAC4-Anti) plants after 2 weeks of N-deficiency treatment; **(B)** Total chlorophyll content in apple leaves; **(C)** Expression levels of chlorophyll metabolism-related genes (*MdNAC4*, *MdNYC1*, *MdPAO*, *MdSGR1*, *MdHEMA*, *MdCHLI*, and *MdCHLM*). pIR: IL60-1 + IL60-2; MdNAC4-OE: IL60-1 + MdNAC4-IL60-2; TRV: TRV1 + TRV2; MdNAC4-Anti: TRV1 + MdNAC4-TRV2. The data are expressed as the means ± SDs (*n* = 3). The significance of the differences was tested by one-way ANOVA; *p* < 0.05.

Leaf senescence is often accompanied by the degradation of chlorophyll. To investigate the biological function of *MdNAC4* in the regulation of leaf senescence, transient transgenic apple leaves were generated (Figure 2A). The MdNAC4-pIR construct was used to generate MdNAC4-OE lines, and the MdNAC4-TRV construct was used to generate MdNAC4-antisense (-Anti) lines. Apple leaves were infected *via* vacuum infiltration, and non-transgenic apple leaves in the same period and at the same leaf position were used as controls. The qRT-PCR results showed that the expression level of *MdNAC4* in the leaves infected with MdNAC4-pIR was significantly higher than that in the controls and significantly lower in the leaves infected with MdNAC4-TRV (Figure 2C), indicating that *MdNAC4* was successfully injected into apple leaves. The infected leaves were placed on N-deficient plates, and the leaf phenotypes were observed under long-day conditions (14 h:10 h; light/darkness; 22°C). The results showed that the apple leaves overexpressing *MdNAC4* showed a significant senescence phenotype and less chlorophyll content, while the apple leaves in which *MdNAC4* was silenced showed a weakly senescent phenotype and higher chlorophyll content (Figures 2A,B). Leaf senescence is often accompanied by chlorophyll degradation, so we measured the expression of chlorophyll metabolic pathway genes by qRT-PCR. The results showed that overexpression of *MdNAC4* upregulated the expression of chlorophyll catabolism-related genes (*MdNYC1*, *MdPAO*, and *MdSGR1*) and downregulated the expression of chlorophyll synthesis pathway genes (*MdHEMA*, *MdCHLM*, and *MdCHLI*; Figure 2C). These results indicate that *MdNAC4* may regulate apple leaf senescence through the chlorophyll metabolism pathway.

### MdNAC4 Directly Regulates the Expression of the CCGs *MdNYC1* and *MdPAO*

**Figure 3:**
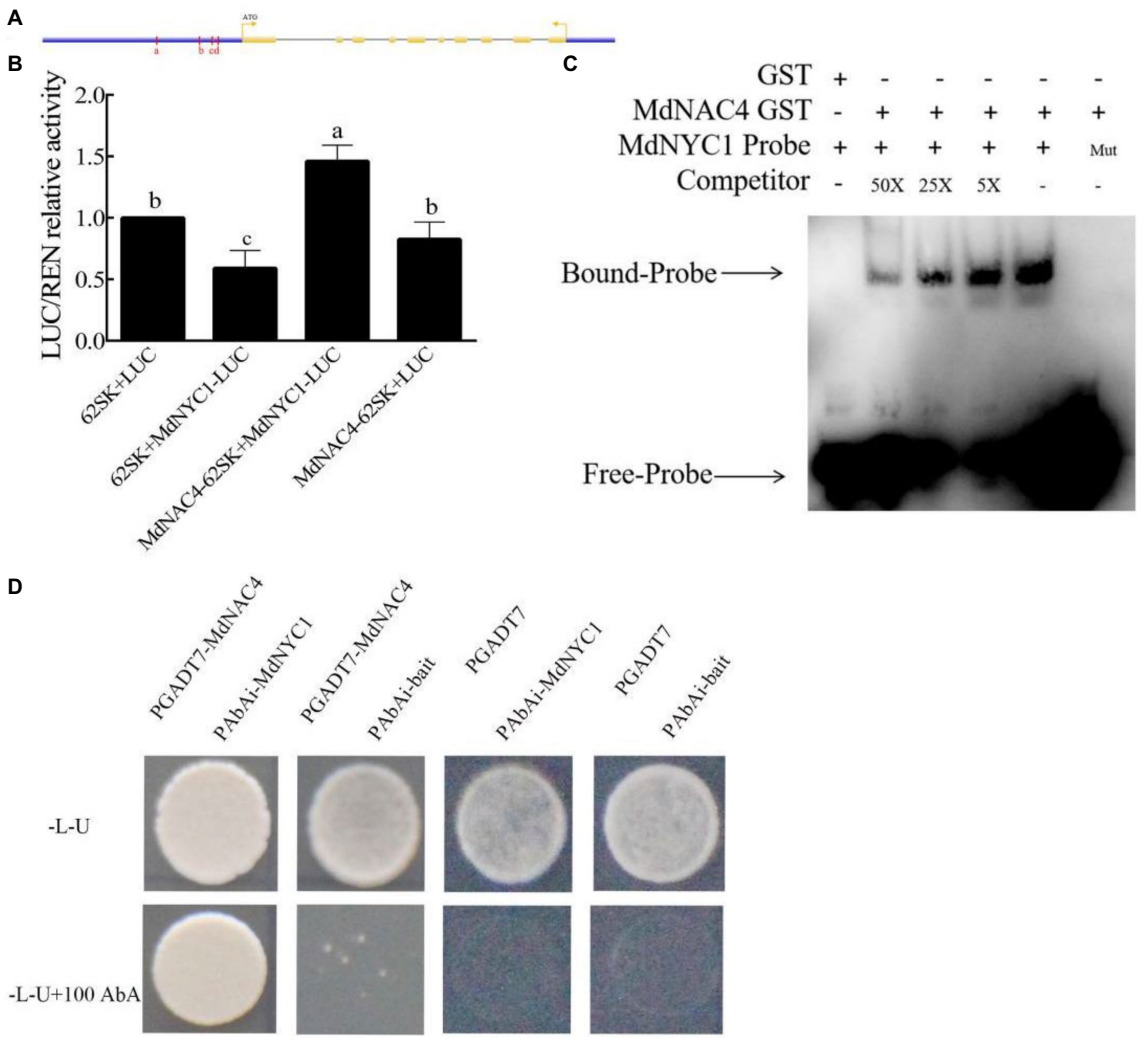
MdNAC4 activated the expression of *MdNYC1*. **(A)** Diagram of the *MdNYC1* gene promoter region. a-d, represent the potential sites to which MdNAC4 might bind. **(B)** Transient expression assay of tobacco leaves showing that MdNAC4 activated the expression of *MdNYC1*. **(C)** EMSA showing that the MdNAC4-GST fusion protein was bound to the MdNYC1 promoter. Unlabeled probes were used as competitors, with “Mut” representing the mutated probe in which the 5’-ACGTG-3′ motif was replaced by 5’-CCGTC-3′. **(D)** Y1H assay revealing the interaction between MdNAC4 and the *MdNYC1* promoter. The data are expressed as the means ± SDs (*n* = 3). The significance of the differences was tested by one-way ANOVA; *p* < 0.05.

**Figure 4:**
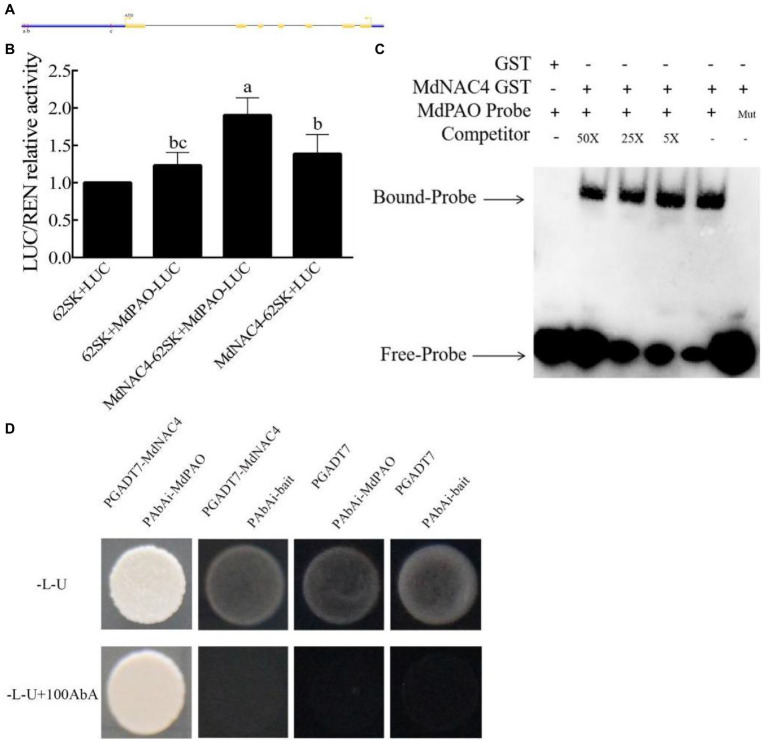
MdNAC4 activated the expression of *MdPAO*. **(A)** Diagram of the *MdPAO* gene promoter region. a-c, represent the potential sites to which MdNAC4 might bind. **(B)** Transient expression assay of tobacco leaves showing that MdNAC4 activated the expression of *MdPAO*. **(C)** EMSA showing that the MdNAC4-GST fusion protein was bound to the *MdPAO* promoter. Unlabeled probes were used as competitors, with “Mut” representing the mutated probe in which the 5’-ACGTG-3′ motif was replaced by 5’-CCGTC-3′. **(D)** Y1H assay revealing the interaction between MdNAC4 and the *MdPAO* promoter. The data are expressed as the means ± SDs (*n* = 3). The significance of the differences was tested by one-way ANOVA; *p* < 0.05.

Considering the promoting role of MdNAC4 in leaf senescence and chlorophyll degradation, we speculated that MdNAC4 may directly regulate the expression of genes related to chlorophyll catabolism. To explore the mechanism by which MdNAC4 regulates the expression of CCGs, we analyzed the promoter region of *NYC1*, *PAO*, and *SGR* including the region 2 kb upstream of the start codon. There were seven ABRE cis-acting elements found in the *NYC1* promoter region, which were grouped into four regions (a, b, c, d) based on their distance (Figure 3A). Three ABRE cis-acting elements identified in the *PAO* promoter region could be further grouped according to three regions (a, b, c; Figure 4A). An ABRE cis-acting element identified in the *SGR* promoter region (Supplementary Figure 1A). To confirm the regulatory relationships between MdNAC4 and *MdNYC1*, *MdPAO*, and *MdSGR*, we established a transient dual effector-reporter system in tobacco leaves. The MdNAC4 CDS were fused to pGreenII 62-SK to generate an effector construct. The *MdNYC1*, *MdPAO*, and *MdSGR* promoter fragments were fused to the pGreenII 0800-LUC vector to generate a reporter construct. Tobacco leaves were injected with different *Agrobacterium* strain mixtures (62SK + LUC, 62SK + MdNYC1 pro-LUC, MdNAC4-62SK + MdNYC1 pro-LUC, MdNAC4-62SK + LUC). It was clear that the LUC activity of tobacco co-injected with the MdNAC4-62SK and MdNYC1 pro-LUC was higher than that of the control groups (Figure 3B). Similarly, the LUC activity of tobacco co-injected with the MdNAC4-62SK and MdPAO pro-LUC was also higher than that of the control groups (Figure 4B). However, the LUC activity of tobacco co-injected with the MdNAC4 and *MdSGR* promoters was no significant difference with the control groups (Supplementary Figure 1C). This indicates that MdNAC4 directly activated the reporter driven by the promoters of *NYC1* and *PAO*, but did not activate the reporter driven by the promoter of *SGR*.

To further confirm the binding of the MdNAC4 proteins to these regions, an integrated EMSA assay was performed. As shown in Supplementary Figures 2, 3, MdNAC4-GST fusion protein could bind to these regions. In addition, the MdNAC4-GST fusion protein could specifically bind the *MdNYC1* and *MdPAO* probes (ABRE 5′-ACGTG-3′) but not the mutant probe (5′-CCGTC-3′; Figures 3C, 4C). When different concentrations of competing probes were added, the binding bands became weaker, and this binding was lost with the addition of the mutation probe. Alternatively, Y1H assays were performed to confirm the interaction between the MdNAC4 protein and the *MdNYC* and *MdPAO* promoters. The full-length *MdNAC4* gene and the promoter fragments of *MdNYC* and *MdPAO* containing ABRE cis-acting elements were cloned into the pGADT7 and pAbAi vectors, respectively. The resulting pGADT7-MdNAC4 and pAbAi-MdNYC, pAbAi-PAO fusion plasmids were co-transferred into Y1H yeast competent cells. The positive cloned strains grew normally on the selected media, but the growth of the empty vector was inhibited (Figures 3D, 4D), indicating that there was a direct interaction between MdNAC4 and *MdNYC1*, *MdPAO*, respectively. Taken together, these results suggest that MdNAC4 can directly bind the promoters of *MdNYC1* and *MdPAO* and activate their expression.

### MdNAC4 Interacts With MdAPRR2

To further explore the mechanism by which MdNAC4 regulates leaf senescence, a Y2H assay was used to screen for proteins interacting with MdNAC4 from the apple leaf cDNA library. First, we detected the auto-activation activity of the MdNAC4 protein and found that full-length MdNAC4 has auto-activation activity (Supplementary Figure 4). We then divided the full-length MdNAC4 protein into three regions: amino acids 1–146, amino acids 147–285, and amino acids 286–350. Among them, 1–146 aa contains a NAM conserved domain with slight auto-activation activity, 147–285 aa without self-activation activity, and 286–350 aa also has auto-activation activity. Finally, 147–285 aa regions were selected to screen the library.

**Figure 5:**
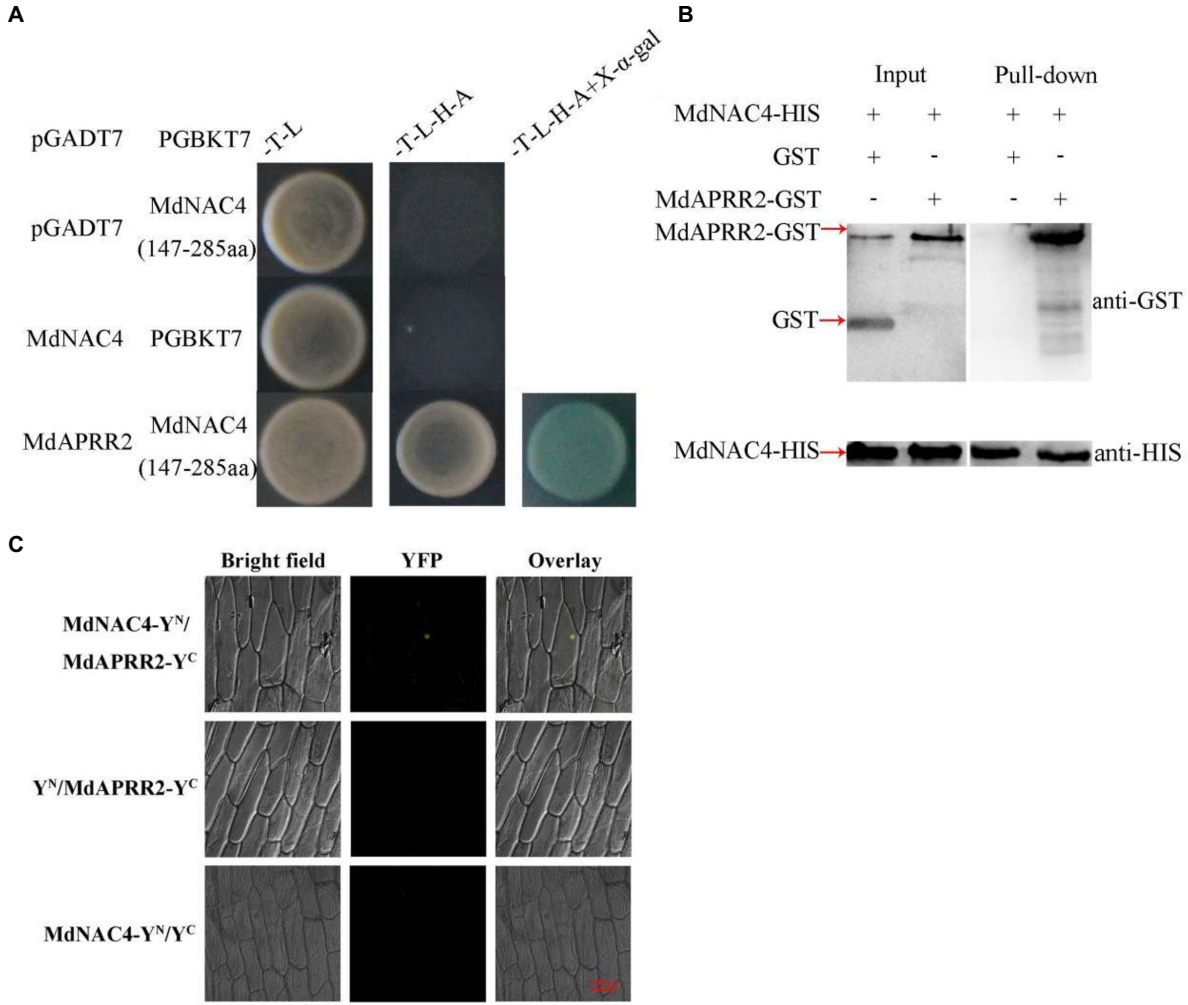
MdNAC4 interacted with MdAPRR. **(A)** Interaction between MdNAC4 and MdAPPRR2 *via* a Y2H assay; **(B)** Interaction between MdNAC4 and MdAPPRR2 *via* a pull-down assay; **(C)** Interaction between MdNAC4 and MdAPPRR2 *via* a BiFC assay.

According to the results of the Y2H screening library (Supplementary Table 2), we found that the ARR-B TF MdAPRR2 interacted with MdNAC4. To further confirm that MdNAC4 interacts with the MdAPRR2 protein, we tested by Y2H experiments the interactions of MdNAC4 with MdAPRR2 using pGBKT7-MdNAC4 (147–285 aa) as bait, pGADT7-MdAPRR2 as prey and empty vector as controls. The results of the Y2H experiment showed that only yeast strains cotransformed with MdNAC4 and MdAPRR2 were able to grow on SD/-T/-L/-H/-A media, while the growth of yeast strains transformed with the empty vectors was inhibited (Figure 5A). These findings indicate that there is an interaction between MdNAC4 and MdAPRR2 proteins in yeast cells.

In addition, we carried out a pull-down assay *in vitro*. The MdAPRR2-GST fusion protein could be pulled down by the MdNAC4-HIS protein, but the GST protein could not, which indicated that MdNAC4 interacts with MdAPRR2 *in vitro* (Figure 5B).

Finally, a bimolecular fluorescence complementation (BiFC) assay was performed to verify the interaction between MdNAC4 and MdAPRR2 (Figure 5C). The MdNAC4 protein and the MdAPRR2 protein were fused to the N-terminal (pSPYNE) and C-terminal (pSPYCE) of yellow fluorescent protein, respectively. The recombinant MdNAC4-YFP^N^ and MdAPRR2-YFP^C^ vectors were transformed into onion epidermal cells. The fluorescence detection results indicated that MdNAC4 interacts with MdAPRR2. In summary, these results suggest that MdNAC4 interacts with MdAPRR2 proteins *in vivo* and *in vitro*.

### Overexpression of *MdAPRR2* Delays Leaf Senescence Induced by N Deficiency

**Figure 6:**
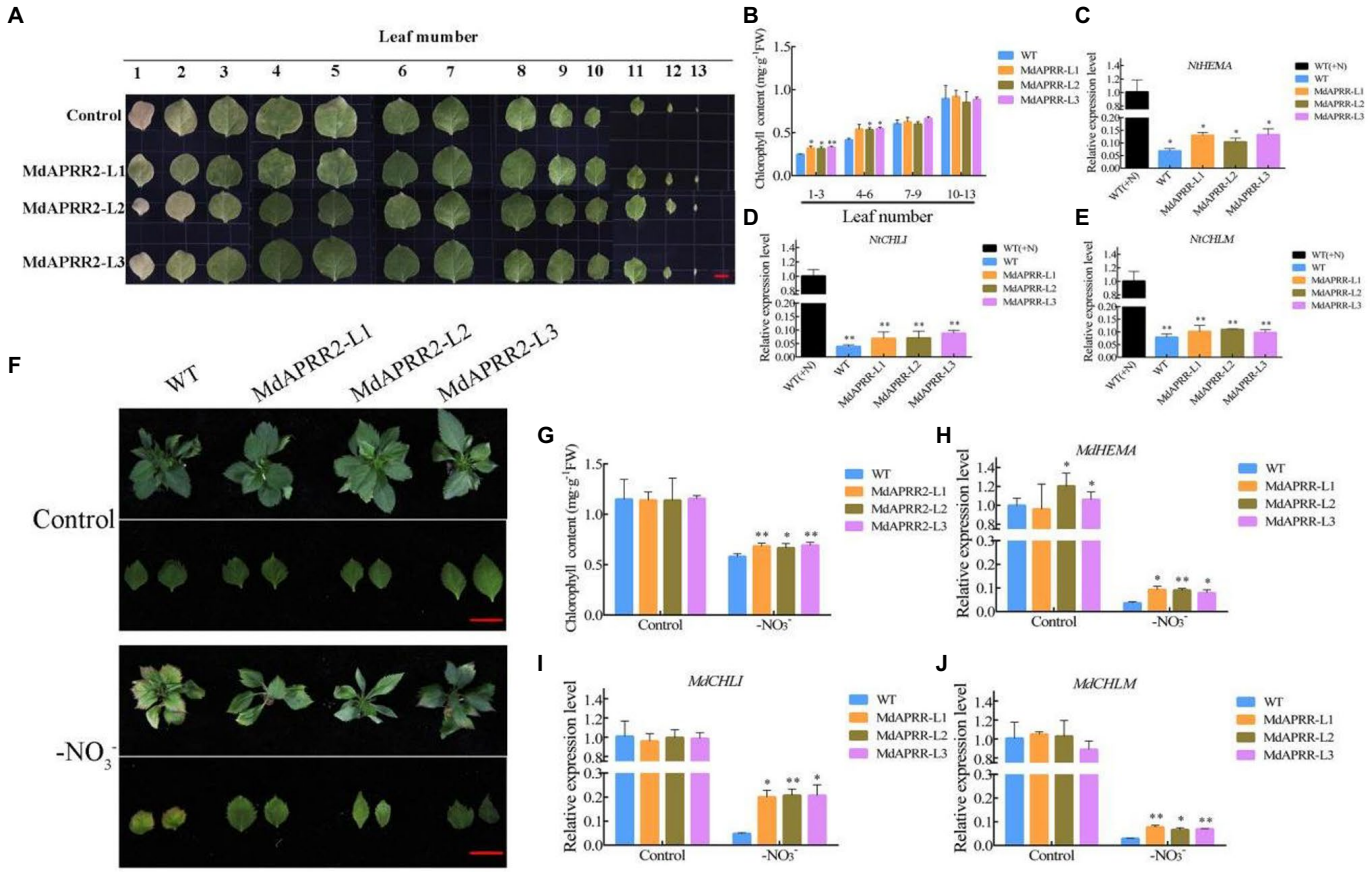
Overexpression of *MdAPRR2* delayed chlorophyll degradation induced by N deficiency. **(A)** Phenotypes of WT (Control) and transgenic tobacco (*MdAPRR2*) leaves after 3 weeks of N-deficiency treatment; **(B)** Total chlorophyll content in tobacco leaves indicated in **(A)**; **(C–E)** Expression levels of the chlorophyll synthesis-related genes *MdHEMA*, *MdCHLI*, and *MdCHLM* in tobacco; **(F)** Phenotypes of WT and transgenic apple (*MdAPRR2*) leaves after 4 weeks of N-deficiency treatment; **(G)** Total chlorophyll content in tobacco leaves indicated in **(F)**; **(H–J)** Expression levels of the chlorophyll synthesis-related genes *MdHEMA*, *MdCHLI*, and *MdCHLM* in apple seedlings. The data are expressed as the means ± SDs (*n* = 3). Significant differences were detected by a *t*-test: ^*^p < 0.05 and ^**^p < 0.01.

Considering that MdAPRR2 interacts with MdNAC4 and that MdNAC4 positively regulates leaf senescence induced by N deficiency, MdAPRR2 may also be involved in leaf senescence induced by N deficiency. First, stable transgenic tobacco and apple plants were generated (Supplementary Figure 5). Four-week-old tobacco was cultured for 3 weeks under N deficiency. The results showed that MdAPRR2-OE lines exhibited a delayed senescence phenotype, and the chlorophyll content of leaves 1–3 and 4–6 was significantly higher than that of the controls (Figures 6A,B). In addition, we measured the expression of chlorophyll synthesis-related genes (*NtHEMA*, *NtCHLI*, and *NtCHLM*) in tobacco by qRT-PCR. The results showed that the expression levels of chlorophyll synthesis-related genes in both transgenic and wild-type (WT) tobacco decreased after N-deficiency treatment, but the decrease was more significant in WT tobaccos (Figures 6C–E). Similarly, compared with the WT controls, MdAPRR2-OE transgenic apple seedlings showed a weaker senescence phenotype and higher chlorophyll content after N-deficiency treatment (Figures 6F,G). Consistently, the expression of chlorophyll synthesis-related genes (*MdHEMA*, *MdCHLI*, *MdCHLM*) was significantly higher than that of the controls (Figures 6H–J). Considering the role of MdAPRR2 in chlorophyll metabolism, we measured the expression levels of CCGs in MdAPRR2-OE lines (Supplementary Figure 6). The results showed that the expression levels of CCGs in both transgenic and wild-type (WT) apple seedlings increased after N-deficiency treatment, but the increase was more significant in WT apple seedlings.

In addition, the chloroplasts in apple leaves were observed *via* transmission electron microscopy (Supplementary Figure 7). No significant difference was found between *MdAPRR2* overexpression and WT plants before treatment. The chloroplast morphology was changed, the chlorophyll membrane was degraded, the grana thylakoid was bent, and the stromal lamella was reduced after N-deficiency treatment. Furthermore, the number of starch granules in MdAPRR2-OE cells was significantly higher than that in WT control cells, and there were more stroma lamellae. These observations suggest that MdAPRR2 may act as a negative regulator of leaf senescence.

## Discussion

Leaves are the main photosynthetic organs used to harvest energy and produce carbohydrates during growth and development (Woo et al., 2019). When leaves enter the senescence stage, catabolic activity increases, and metabolism and gene expression change orderly. The major transitions in the process of leaf senescence are chloroplast degradation, chlorophyll breakdown, and the reallocation of cellular materials that accumulated during vegetative growth from senescent leaves to younger tissues (Gregersen et al., 2008). Thus, leaf senescence is an important process to ensure crop yields and increased survival (Buchanan-Wollaston et al., 2003; Lim et al., 2007). Apple (*M. domestica*) is a perennial deciduous fruit tree species whose leaf life has a great impact on fruit yield and quality. Premature leaf senescence reduces the time for photosynthesis and carbon assimilation, which eventually leads to a reduction in yield and poor quality (An et al., 2019). It is therefore highly important to study the metabolic pathway and regulatory network of leaf senescence with respect to understanding the growth and development of fruit trees and promoting apple production.

Leaf senescence is a complex and strictly regulated biological process influenced by internal factors and external environmental stresses (Woo et al., 2019). The senescence process is accompanied by a large number of senescence-associated gene (SAG) expression changes. Previous studies have shown that NAC TFs are key factors regulating transcriptional changes in leaf senescence (Breeze et al., 2011; Woo et al., 2019). *Arabidopsis* NTL4 (Lee et al., 2012), *Medicago truncatula* NAC969 (de Zélicourt et al., 2012), wheat GPC (Distelfeld et al., 2012), *Populus* RD26/ANAC072 (Wang et al., 2021), and other NAC TFs regulate leaf senescence by activating the transcriptome of SAGs. In addition, NAC TFs are also involved in nutrient stress-induced leaf senescence. The *Arabidopsis* NAC TF ORE1 is considered to be the main factor in N deficiency-induced leaf senescence, and the NLA gene delays N deficiency-induced leaf senescence by reducing the abundance of the NAC TF ORE1, while UBP12 and UBP13 accelerate N deficiency-induced leaf senescence by increasing the level of ORE1 protein (Park et al., 2018, 2019). These results suggest that NAC TFs may play positive regulatory roles in N deficiency-induced leaf senescence.

In the process of leaf senescence, chlorophyll degradation is the most characteristic, and the degradation of chlorophyll is an indicator of leaf senescence (Mao et al., 2017). The degradation of chlorophyll is mainly governed by the pheophorbide an oxygenase (PAO)/phycobilin pathway, which includes a series of CCGs (Hörtensteiner and Kräutler, 2011). Previous studies have shown that NAC TFs directly bind to a series of CCGs promoters to regulate leaf senescence. *Arabidopsis* NAC TF ANAC046 directly binds to the promoters of the CCGs *NYC1*, *SGR1*, *SGR2*, and *PAO* to positively regulate chlorophyll catabolism (Oda-Yamamizo et al., 2016). The rice NAC TF NAC2 directly interacts with the promoter sequences of the CCGs *SGR* and *NYC3* to promote chlorophyll catabolism (Mao et al., 2017). Additionally, other NAC TFs, such as NAP, NTL4, and NAC106, also participate in leaf senescence by directly controlling the expression of CCGs (Lee et al., 2012; Liang et al., 2014; Sakuraba et al., 2015). Although substantial progress has been made in the regulation of leaf senescence by NAC TFs, the molecular mechanism through which NAC TFs regulate N deficiency-induced leaf senescence remains unclear.

In this study, the NAC TF MdNAC4, which acts as an important regulator in N deficiency-induced leaf senescence, was identified and characterized. Overexpression of *MdNAC4* caused early senescence induced by N deficiency, suggesting that MdNAC4 may be a positive regulator of leaf senescence under those conditions. The measurement of chlorophyll content and the expression of genes related to the chlorophyll metabolic pathway provided evidence for further study (Figure 2). The chlorophyll content of *MdNAC4* overexpression apple leaves was significantly lower than that of WT control leaves after N-deficiency treatment. The expression of chlorophyll synthesis-related genes was downregulated, and chlorophyll catabolism-related genes was upregulated in *MdNAC4* overexpression apple leaves. Furthermore, the LUC activity of tobacco co-injected with the MdNAC4-62SK and MdNYC1 pro-LUC was higher than that of the control groups. Similarly, the LUC activity of tobacco co-injected with the MdNAC4-62SK and MdPAO pro-LUC was also higher than that of the control group. Since only when MdNAC4 coexisted with MdNYC1 or MdNAC4 coexisted with MdPAO, the high expression of LUC activity in tobacco leaves could be induced. The results suggest that MdNAC4 activates the expression of *MdNYC1* and *MdPAO*. In addition, the results of EMSA and Y1H assays also showed that MdNAC4 directly interacts with the promoter of the CCGs *MdNYC1* and *MdPAO* (Figures 3, 4). Therefore, MdNAC4 may be a new N deficiency-induced leaf SAG and that MdNAC4 may mediate the chlorophyll metabolic pathway to influence leaf senescence.

Changes in leaf color are mainly caused by the degradation of chlorophyll. Brand et al. revealed that the chlorophyll content of pepper fruits was mainly controlled by two QTLs-GLK2 and *Arabidopsis* pseudo-response regulator 2-like (APRR2; Brand et al., 2012, 2014). Previous studies have shown that the GLK2 TF can coregulate and synchronize the expression of light capture and chlorophyll synthesis genes and that overexpression of *MdGLK2* results in increased chlorophyll accumulation and delayed leaf senescence (Pan et al., 2013; Song et al., 2014; Jeong et al., 2020). APRR2 is closely related to fruit pigment accumulation, and overexpression of *MdAPRR2* significantly was shown to increase the chlorophyll content compared with that of the controls (Pan et al., 2013). In addition, APRR2 can interact with the calmodulin-like protein CLM9, which is involved in tolerance to abiotic stress (Perochon et al., 2010). APRR2 can also interact with the ABSCISIC ACID INSENSITIVE 3 (ABI3) TF, which is involved in promoting the expression of genes containing ABA-responsive elements (Kurup et al., 2000). These results indicated that APRR2 may be involved in chlorophyll metabolism mediated by abiotic stress. In the present study, MdAPRR2 was shown through a series of biochemical assays to interact with MdNAC4 proteins (Figure 5). The results shown in Figure 6 indicate that the function of MdAPRR2 in the N deficiency-induced leaf senescence process was opposite to that of MdNAC4. Furthermore, both the *MdAPRR2* overexpression and WT plants showed leaf yellowing and chlorophyll loss, while overexpression of *MdAPRR2* helped prevent the senescence phenotype and chlorophyll loss induced by N deficiency. The expression of chlorophyll synthesis-related genes in the MdNAC4*-*OE lines was higher than that in the controls. Therefore, MdAPRR2 may promote chlorophyll synthesis by activating the expression of chlorophyll synthesis-related genes and thus delay leaf senescence induced by N deficiency.

**Figure 7:**
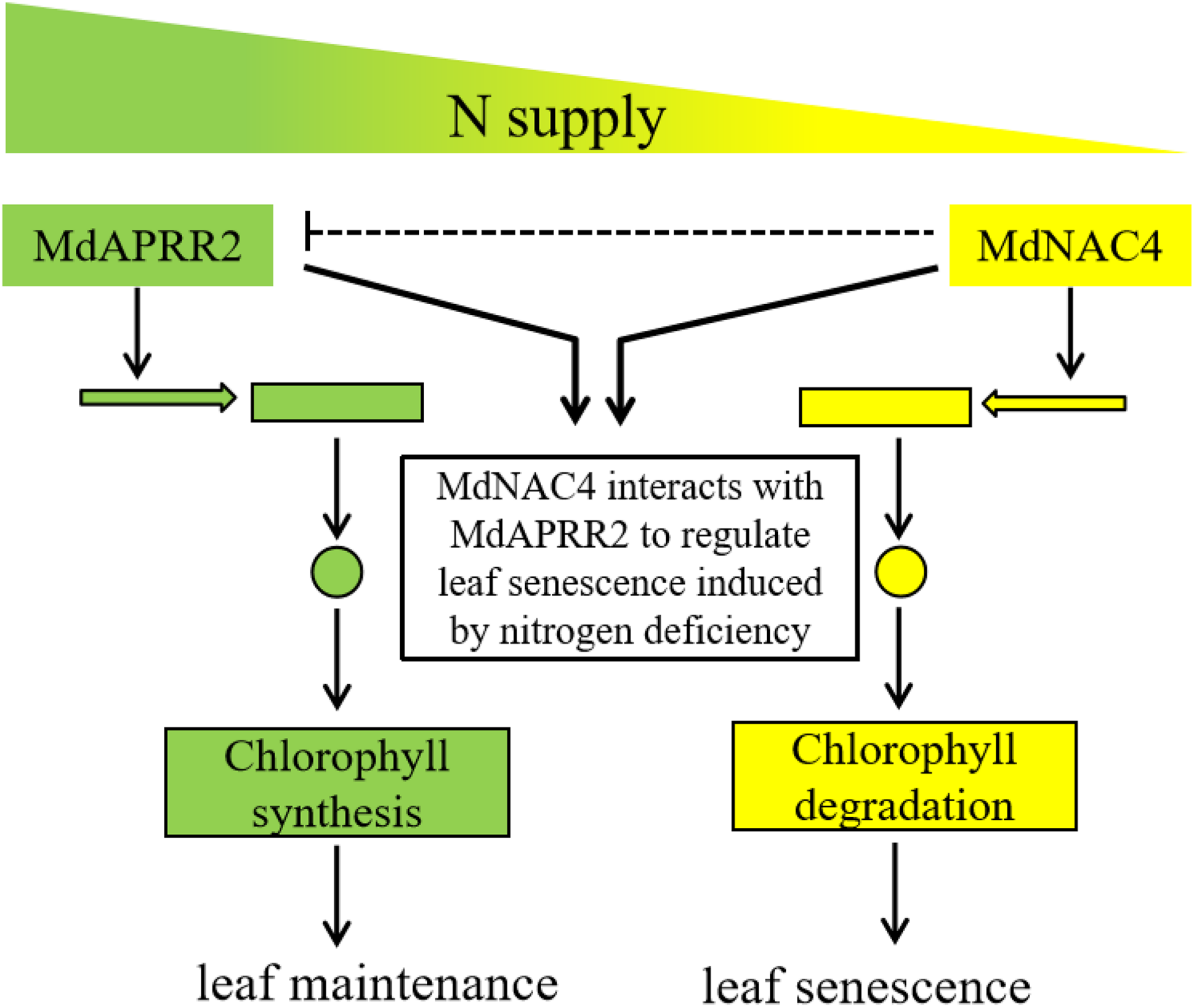
Working model of MdNAC4 regulating N deficiency-induced leaf senescence. The expression of CCGs (yellow) is upregulated, while the expression of chlorophyll synthesis genes (green) is downregulated under nitrogen-deficient conditions. The expression of MdAPRR2 target genes (green) is downregulated in the presence of MdNAC4 protein, which leads to the inhibition of chlorophyll synthesis, while the expression of MdNAC4 target genes (yellow) is upregulated, which accelerates chlorophyll catabolism.

In summary, based on our current study, a model was proposed to explain how MdNAC4 and MdAPRR2 interact to regulate N deficiency-induced leaf senescence (Figure 7). MdNAC4 binds to CCGs (*MdNYC1* and *MdPAO*) promoters and activates their expression, thus accelerating N deficiency-induced leaf senescence. On the other hand, MdAPRR2 delays N deficiency-induced leaf senescence by upregulating the expression of genes related to chlorophyll synthesis. MdNAC4 interacts with MdAPRR2 to form an inactive heterodimer, which activates the expression of the MdNAC4 target gene, while the expression of MdAPRR2 target genes is inhibited. Thus, the yellowing of leaves induced by N deficiency are mainly caused by MdNAC4. It seems that apple may employ a balancing mechanism to regulate N deficiency-induced leaf senescence. Our findings provide a new insight for studying the molecular mechanism of leaf senescence induced by N deficiency.

## Data Availability Statement

The original contributions presented in the study are included in the article/Supplementary Material, further inquiries can be directed to the corresponding authors.

## Author Contributions

LL and BW designed the research. WX and XC contributed new models. BW wrote the manuscript. XG and QT revised the intellectual content of the manuscript. WZ collected the plant materials and extracted RNA. DL supervised the expression and data analysis. All authors contributed to the article and approved the submitted version.

## Funding

This study was funded by Natural Science Foundation of Shandong Province (ZR2020ZD18), Shandong Provincial Fruit Industry Technology System – Cultivation and Soil Fertilization Post (SDAIT-06-04), Shandong Province Major Science and Technology Innovation Project (2018CXGC0209).

## Conflict of Interest

The authors declare that the research was conducted in the absence of any commercial or financial relationships that could be construed as a potential conflict of interest.

## Publisher’s Note

All claims expressed in this article are solely those of the authors and do not necessarily represent those of their affiliated organizations, or those of the publisher, the editors and the reviewers. Any product that may be evaluated in this article, or claim that may be made by its manufacturer, is not guaranteed or endorsed by the publisher.
