# Supplementary material for: MdNAC4 Interacts With MdAPRR2 to Regulate Nitrogen Deficiency-Induced Leaf Senescence in Apple (Malus domestica)
Source: Front Plant Sci. 2022 Jun 30;13:925035. doi: 10.3389/fpls.2022.925035 (PMC9280364; doi:10.3389/fpls.2022.925035)
Supplement: Supplementary file 1 [file Data_Sheet_1.docx]

**
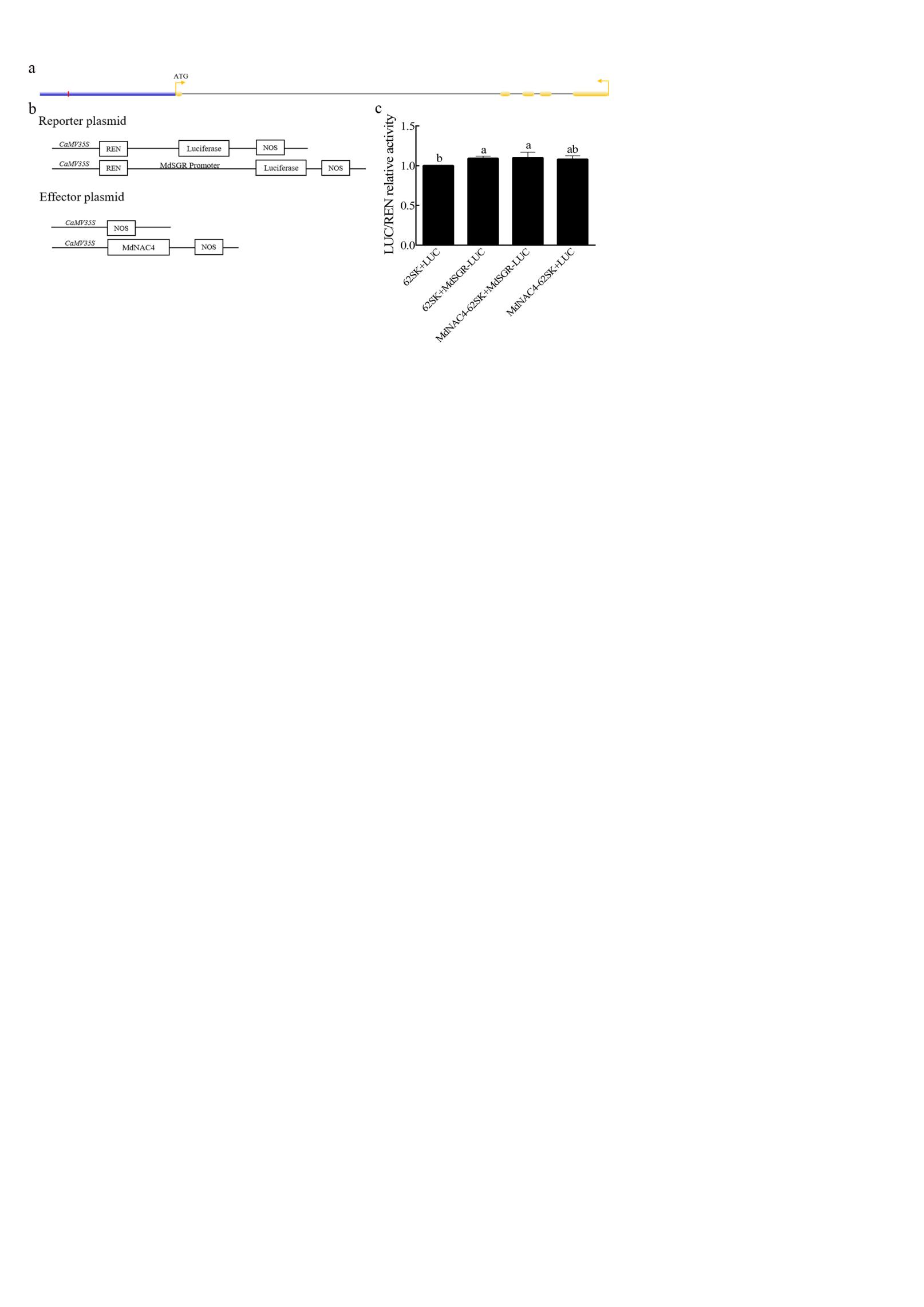
**

**Supplementary Figure 1 MdNAC4 indirectly activated the expression of MdSGR.**

(**a**) Diagram of the MdSGR gene promoter region. (**b**) Reporter and effector constructs used in the transient assay. (**c**) LUC/REM relative activity. An empty vector was used as the control. Data are expressed as the means ± SD, n = 3. The different letters denote significant differences according to one-way analysis of variance (ANOVA) (P < 0.05).

**
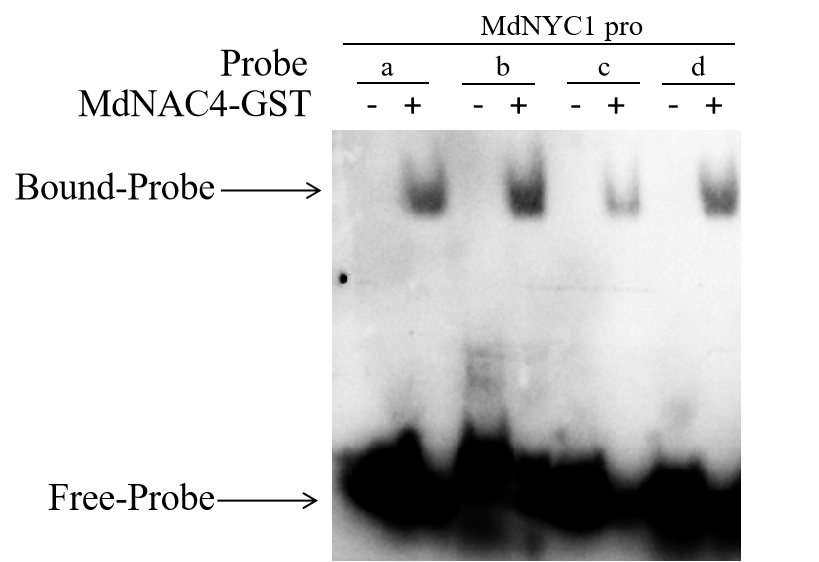
**

**Supplementary Figure 2 MdNAC4 binds to the promoter of MdNYC1.
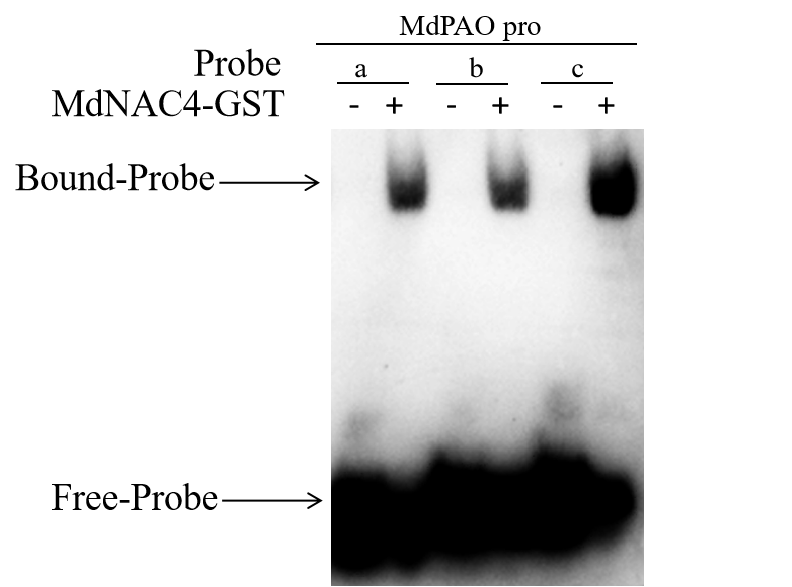
**

**Supplementary Figure 3 MdNAC4 binds to the promoter of MdPAO.**

**
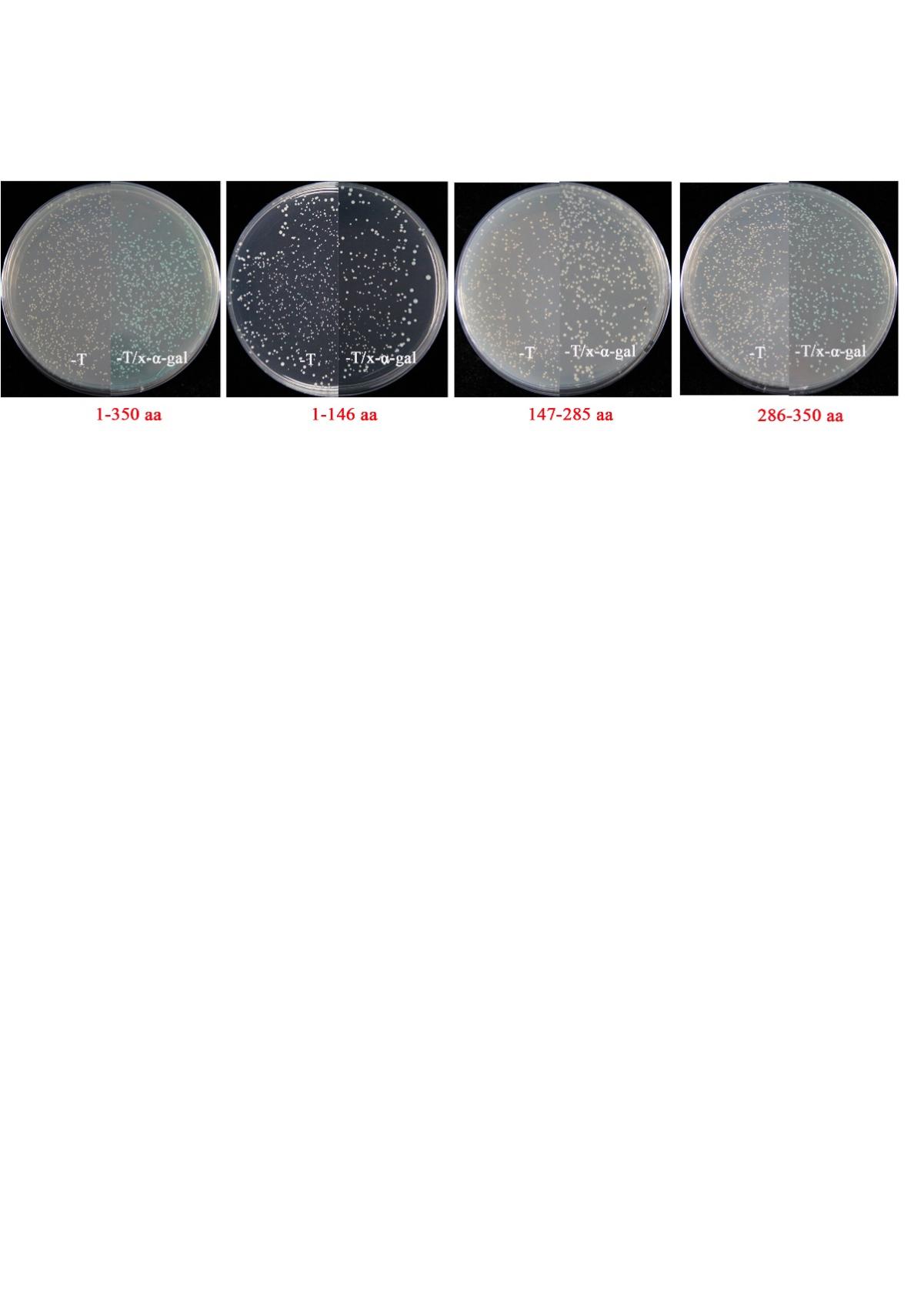
**

**Supplementary Figure 4 Auto-activation verifications of MdNAC4.**

**
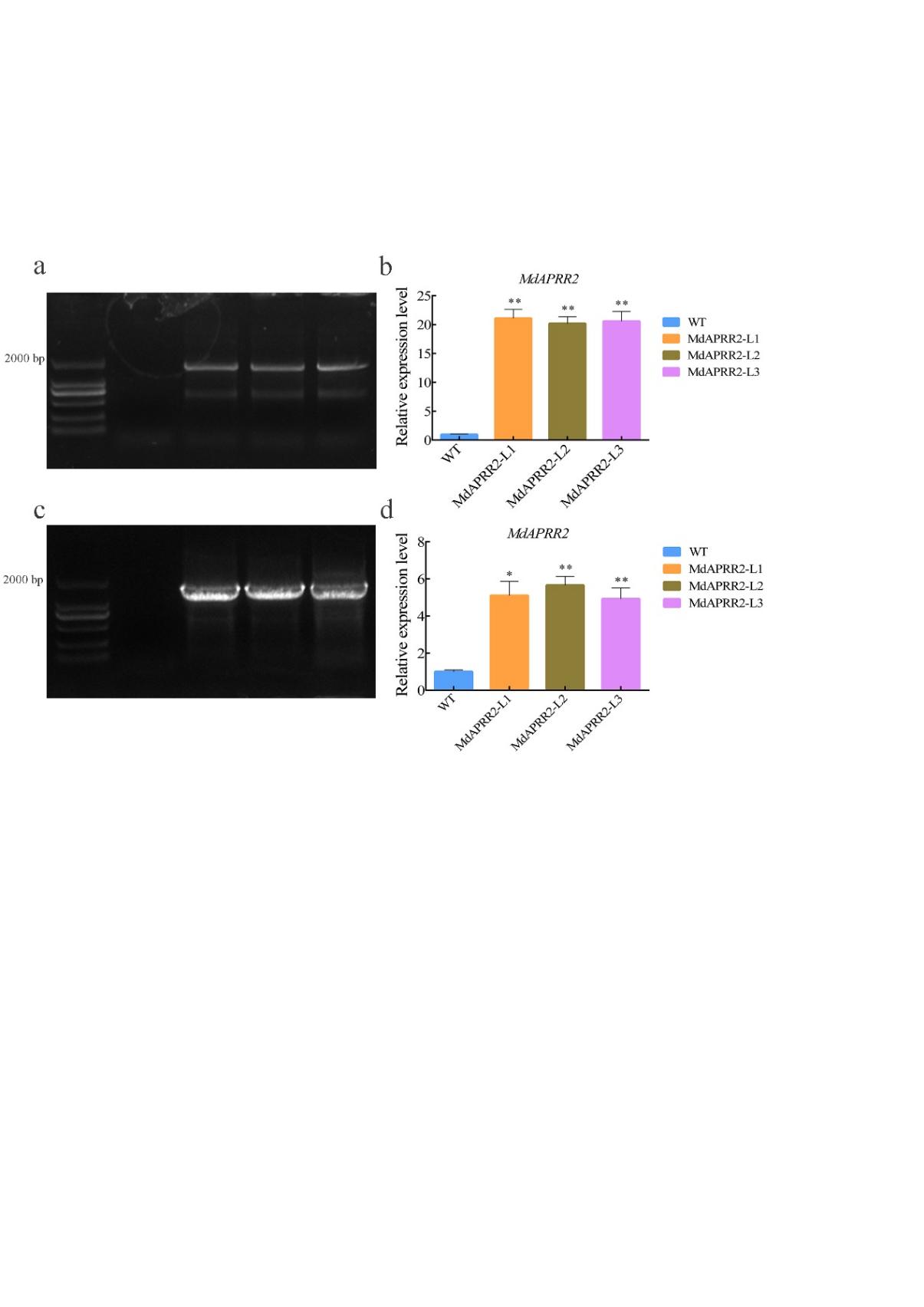
**

**Supplementary Figure 5 Identification of transgenic tobacco and apple seedlings overexpressed MdAPRR2.**

Identification of transgenic tobacco (a) and apple seedlings (c) overexpressed MdAPRR2 by PCR; Identification of transgenic tobacco (b) and apple seedlings (d) overexpressed MdAPRR2 by qRT-PCR.

**
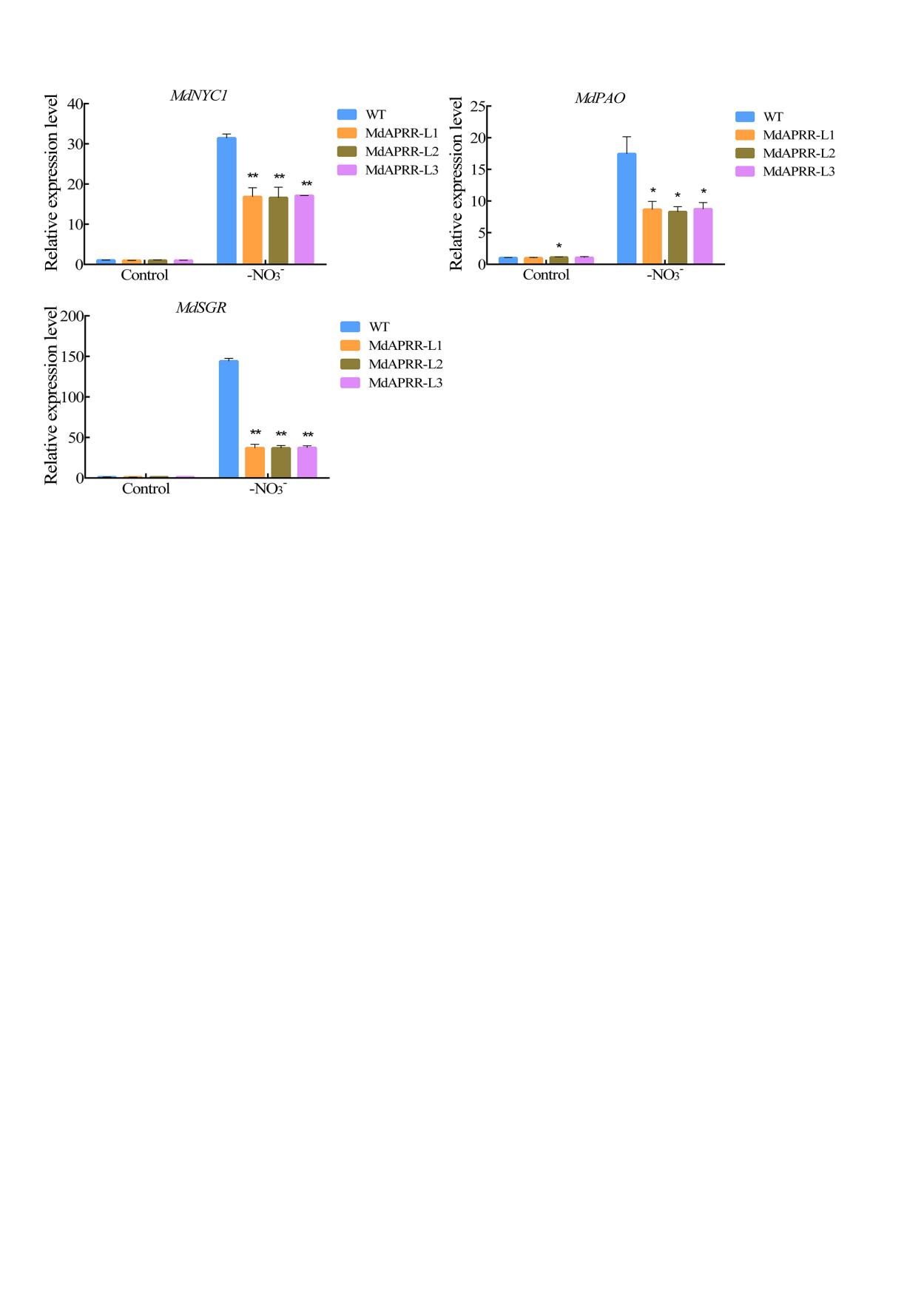
**

**Supplementary Figure 6 Effects of overexpression of *MdAPRR2* on the expression of CCGs (*MdNYC1*, *MdPAO*, and *MdSGR*).**

**
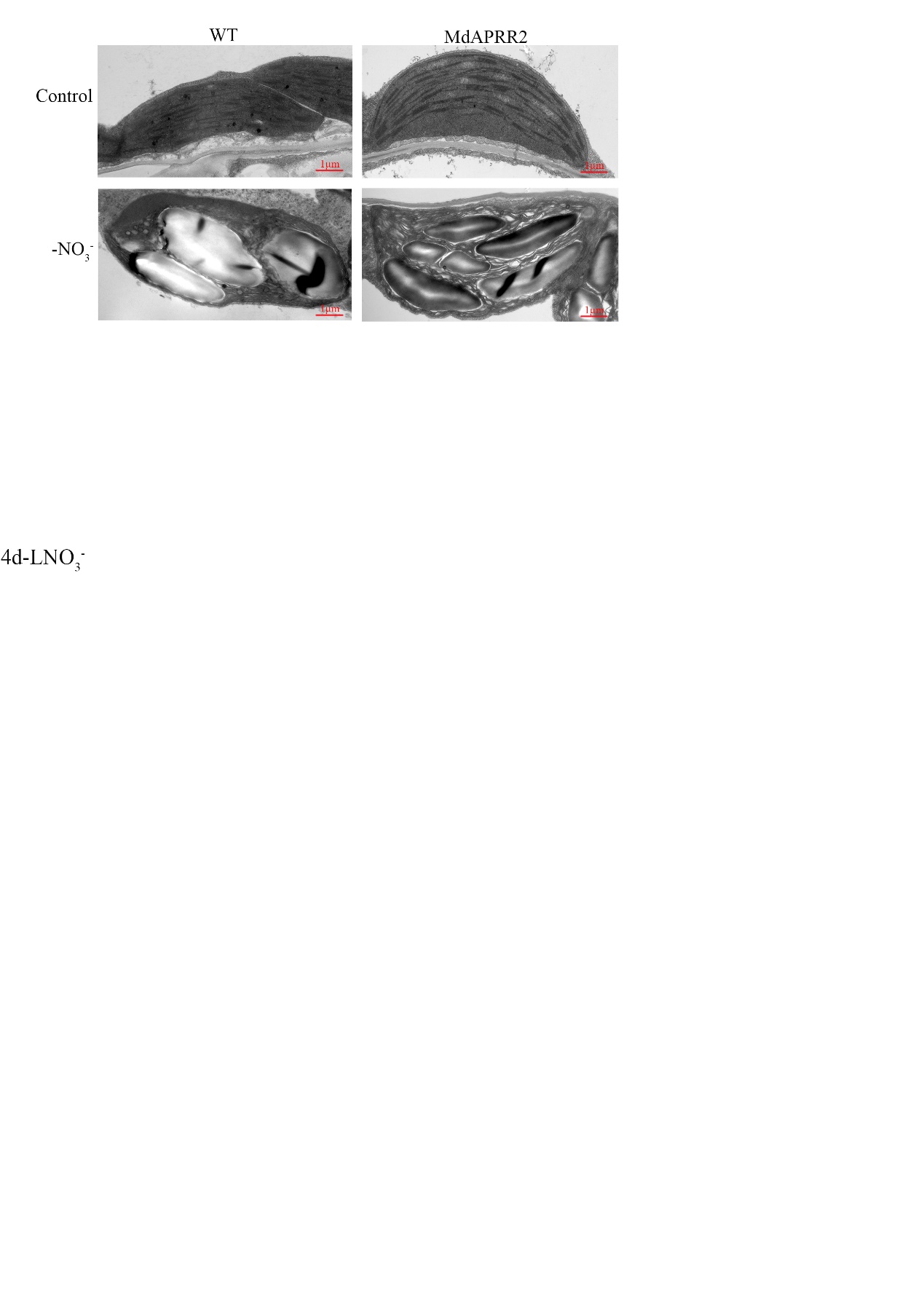
**

**Supplementary Figure 7 The chloroplast morphology of wild-type and transgenic apple (MdAPRR2) seedlings treated with nitrogen deficiency for 4 weeks was observed by**

**transmission electron microscopic.**

**Supplementary Table 1 The primers used in this study**

| **Name** | **Sequences (5’-3’)** | **use** |
| --- | --- | --- |
| MdMAC4-F | ACTTGAGGCGTAGTGCGAAA | qRT-PCR |
| MdNAC4-R | GTTCTATCGGAGCGGACGAG |  |
| MdNYC1-F | TCTTCGGTTTGGGGTGGTTT |  |
| MdNYC1-R | AACGCCTGTGCTCATCATGG |  |
| MdPAO-F | TCGCCCCCTTATCTGAAGGA |  |
| MdPAO-R | TCTTGGAGACCGAACAGCAC |  |
| MdSGR1F | CCATATTTCCTGTTAATGCAAGGCT |  |
| MdSGR1R | AGGTGCTTCTTTTCGTCCACT |  |
| MdHEMA-F | CAAGCTCCTTCATGGTCCGA |  |
| MdHEMA-R | CGTGCATGTTCTCAAGGGTC |  |
| MdCHLI-F | TGAAGGAGATACTGAAAGAGCCC |  |
| MdCHLI-R | TGGTGAAACCTAAATGTGCTGG |  |
| MdCHLM-F | CTGCATTTTGCTCTGATACCGA |  |
| MdCHLM-R | ACGAAGAGGATGAGATTCGTGA |  |
| NtHEMA-F | AGCATGCAATCACTGTCGGA |  |
| NtHEMA-R | TAGCGTGGGAGGATTCAGGA |  |
| NtCHLI-F | GGCAGAGTCAAGGGAGGAAG |  |
| NtCHLI-R | CCTGTTCTTGAGCAGGGTTG |  |
| NtCHLM-F | CGGAGAGTTGTTCCCTGGAC |  |
| NtCHLM-R | GTGGCAATCAGGCCTCTCTT |  |
| OE-MdNAC4-F | ggactctagaggatccccgggATGGAAAATATTCTGGGTTTATTAACG | Genetic transformation |
| OE-MdNAC4-R | ataagggactgaccacccgggTCAATAATTCCAGAGGCAATCGA |  |
| OE-MdAPRR2-F | ggactctagaggatccccgggATGGTTTGCACTGCCAATGAT |  |
| OE-MdAPRR2-R | gctcaccatggtacccccgggTCAAAAGGACAGATGAATGT |  |
| pIR-MdNAC4-F | gcagaatctgaattcgtcgacATGGAAAATATTCTGGGTTTATTAACG |  |
| pIR-MdNAC4-R | cccccacacgtgtggtctagaATAATTCCAGAGGCAATCGAATTC |  |
| TRV-MdNAC4-F | aaggttaccgaattctctagaGATACATATTTCAGGTTTGGTGAGG |  |
| TRV-MdNAC4-R | tgtcttcgggacatgcccgggAACAAGCGTAAGGAATGTCGT |  |
| BK-MdNAC4-F (1-350 aa) | tcagaggaggacctgcatatgATGGAAAATACTTCTGGGTT | Y2H |
| BK-MdNAC4-R (1-350 aa) | tcgacggatccccgggaattcTCAATAATTCCAGAGGCAATC |  |
| BK-MdNAC4 (1-146 aa)-R | tcgacggatccccgggaattcAGAGTTTTTGCCGTCCAATCT |  |
| BK-MdNAC4 (147-285 aa)-F | tcagaggaggacctgcatatgGCCTGTAATCTCCCCCAAA |  |
| BK-MdNAC4 (147-285 aa)-R | tcgacggatccccgggaattcCAAAACTGAGTTTTGGTGCTGC |  |
| BK-MdNAC4 (285-350 aa)-F | tcagaggaggacctgcatatgATGCAGGACCAGTCGTTTATG |  |
| AD-MdNAC4-F | gtaccagattacgctcatatgATGGAAAATACTTCTGGGTT |  |
| AD-MdNAC4-R | atgcccacccgggtggaattcTCAATAATTCCAGAGGCAATC |  |
| AD-MdAPRR2-F | gtaccagattacgctcatatgTATGGTTTGCACTGCCAATGA |  |
| AD-MdAPRR2-R | gtaccagattacgctcatatgATCTAACCCTGTGTATGGGAGCC |  |
| MdNYC1pro-F | cttgaattcgagctcggtaccATATTTTATTTTATTGTCGAT | Y1H |
| MdNYC1pro-R | agcacatgcctcgaggtcgacTTAGTGGGAGGAGAGTGGC |  |
| MdPAOpro-F | cttgaattcgagctcggtaccCATGATGACGTAAATTCGAACTTCA |  |
| MdPAOpro-R | agcacatgcctcgaggtcgacGGCATAGAGAGAGAGAGAGAGAGAGAG |  |
| GST-MdNAC4-F | gatctggttccgcgtggatccATGGAAAATATTCTGGGTTTATTAACG | Prokaryotic expression *in vitro* |
| GST-MdNAC4-R | gatgcggccgctcgagtcgacTCAATAATTCCAGAGGCAATCGA |  |
| HIS-MdNAC4-F | gccatggctgatatcggatccATGGAAAATATTCTGGGTTTATTAACG |  |
| HIS-MdNAC4-R | tgcggccgcaagcttgtcgacTCAATAATTCCAGAGGCAATCGA |  |
| GST-MdAPRR2-F | gatctggttccgcgtggatccAAGACGGTGATTAAAGAGGAAAATG |  |
| GST-MdAPRR2-R | gatgcggccgctcgagtcgacAAAGGACAGATGAATGTTGAAAGAGT |  |
| MdNAC4^NC^-F | agaacacggggggactctagaATGGAAAATACTTCTGGGT | BiFC |
| MdNAC4^NC^-R | gacagtactatcgatggatccATAATTCCAGAGGCAATCGA |  |
| MdAPRR2^NC^-F | agaacacggggggactctagaATGGTTTGCACTGCCAATGA |  |
| MdAPRR2^NC^-R | gacagtactatcgatggatccACCCTGTGTATGGGAGCCGT |  |
| 62SK-MdNAC4-F | cgctctagaactagtggatccATGGAAAATATTCTGGGTTTATTAACG | Dual luciferase assays |
| 62SK-MdNAC4-R | gggccccccctcgaggtcgacTCAATAATTCCAGAGGCAATCGA |  |
| LUC-MdNYC1-F | gggccccccctcgaggtcgacGATATAGGCACGTGAAGGGAGG |  |
| LUC-MdNYC1-R | cgctctagaactagtggatccTGATGACCACACGTTAGGGAAA |  |
| LUC-MdPAO-F | gggccccccctcgaggtcgacTTAATGGAGATCATTTAGTTATCCATA |  |
| LUC-MdPAO-R | cgctctagaactagtggatccTTTTCAATATTTCCTTCATCTGCAA |  |
| p-MdNYC1-F | ATATAGGCACGTGAAGGGAGGACAGAGATG | EMSA |
| p-MdNYC1-R | CATCTCTGTCCTCCCTTCACGTGCCTATAT |  |
| p-MdPAO-F | AGTTATCCATACGTGTAAAACCAAT |  |
| p-MdPAO-R | ATTGGTTTTACACGTATGGATAACT |  |
| Mut-MdNYC1-F | ATATAGGCCCGTCAAGGGAGGACAGAGATG |  |
| Mut-MdNYC1-R | CATCTCTGGCCTGCCTTCACGTGCCTATAT |  |
| Mut-MdPAO-F | AGTTATCCATCCGTCTAAAACCAAT |  |
| Mut-MdPAO-R | ATTGGTTTTAGACGGATGGATAACT |  |

**Supplementary Table 2 Screening results of MdNAC4 yeast two-hybrid**

| **Gene name** | **Function annotation** |
| --- | --- |
| MD14G1060800 | Rhodanese-like domain-containing protein 9, chloroplastic (LOC103454270) |
| MD15G1236600 | Rhiosulfate sulfurtransferase 16, chloroplastic (LOC103415916) |
| MD04G1231800 | 60S ribosomal protein L23 (LOC103434217) |
| MD08G1029100 | Gamma carbonic anhydrase 1, mitochondrial-like (LOC103428643) |
| MD13G1022400 | Protein C2-DOMAIN ABA-RELATED 7-like (LOC103421781) |
| MD16G1269900 | Two-component response regulator-like APRR2 (LOC103440803) |
| MD10G1055500 | Protein FLOURY 1-like (LOC103445007) |
| MD02G1132400 | Snakin-2-like (LOC103407051) |
| MD15G1370700 | 5’-adenylylsulfate reductase-like 4 (LOC103406080) |
| MD09G1038100 | Gibberellin-regulated protein 14-like (LOC103442284) |
| MD07G1227100 | Abscisic acid receptor PYL4 (LOC103432868) |
| MD11G1253300 | 30S ribosomal protein S20, chloroplastic-like (LOC103448715) |
| MD17G1030400 | Chaperone protein dnaJ C76, chloroplastic (LOC103404310) |
| MD10G1271300 | Cyclic phosphodiesterase-like (LOC103446239) |
| MD01G1136400 | Peptidyl-prolyl cis-trans isomerase FKBP20-1 (LOC103433927) |
| MD03G1257800 | Protein SPIRRIG-like (LOC103432298) |
| MD09G1114800 | 1-aminocyclopropane-1-carboxylate oxidase (LOC103411280) |
| MD05G1089900 | Cinnamyl alcohol dehydrogenase 1 (LOC103420099) |
| MD06G1077700 | Salutaridine reductase-like (LOC103437348) |
| MD05G1221000 | Elongation factor 2 (LOC114819169) |
| MD06G1032600 | Ferredoxin C 2, chloroplastic (LOC103409454) |
| MD03G1114500 | Probable catabolite repression protein creC (LOC103447871) |
